# The effect of preeclampsia on adverse maternal outcomes in Sidama region, Ethiopia: a prospective open cohort study

**DOI:** 10.1038/s41598-022-24034-7

**Published:** 2022-11-11

**Authors:** Birhanu Jikamo, Mulat Adefris, Telake Azale, Kassahun Alemu

**Affiliations:** 1 Department of Epidemiology and Biostatistics, Institute of Public Health, College of Medicine and Health Sciences, University of Gondar, Gondar, Ethiopia; 2 Department of Gynecology and Obstetrics Gondar University Hospital, School of Medicine and Health Sciences, University of Gondar, Gondar, Ethiopia; 3 Department of Health Education and Behavioral Sciences, Institute of Public Health, College of Medicine and Health Sciences, University of Gondar, Gondar, Ethiopia; 4 Hawassa University College of Medicine and Health Sciences, Hawassa, Southern Nations Ethiopia

**Keywords:** Health care, Medical research, Risk factors, Signs and symptoms

## Abstract

Ethiopia has made improvements in the reduction of maternal mortality; the high burden of preeclampsia remains a concern in the Sidama region. This study aimed to measure the effect of preeclampsia on adverse maternal outcomes and identify risk factors among women with preeclampsia in Sidama region. A prospective open cohort study was conducted from August 8, 2019, to October 1, 2020. We enrolled a total of 1015 the pregnant women who had preeclampsia and normotensive women at ≥ 20 weeks of gestation and followed them until 42 days after delivery. A log-binomial logistic regression model was used to estimate the incidence of adverse maternal outcomes and its risk factors. There were 276 adverse maternal outcomes observed in the preeclampsia group compared to 154 adverse maternal outcomes in the normotensive group (*P* < 0.001). Women with severe features of preeclampsia had a 43% (aRR = 1.43, 95% CI 1.3–1.58) higher risk for adverse maternal outcomes compared to women without severe features of preeclampsia. Women without severe features of preeclampsia had a 39% (aRR = 1.39, 95% CI 1.2–1.76) higher risk for adverse maternal outcomes compared to women in the normotensive group. More adverse maternal outcomes occurred among women with preeclampsia after controlling for confounders.

## Introduction

Preeclampsia and eclampsia are two of the most common hypertensive disorders of pregnancy (HDPs)^1^. It is the second leading cause of direct maternal death and is directly responsible for 70,000 maternal deaths annually at the global level^2^. In low-and middle-income countries (LMICs), 10–15% of direct maternal mortalities were associated with preeclampsia and eclampsia in 2018^1^. In Ethiopia in 2019, the pooled prevalence of maternal death was 4%^3^.

In Ethiopia, in 2018, the overall pooled prevalence of HDPs was 6.07%^4^. The same study identified variations in the level of HDPs across different geographical areas: the lowest prevalence was observed in Addis Ababa, the capital city of Ethiopia (5.41%), while the Sidama region in southern Ethiopia had a pooled prevalence of 10.13%^4^. Furthermore, in Ethiopia in 2020, the overall pooled prevalence of preeclampsia was 4.74%^5^.

Adverse maternal outcomes were noted in a study conducted in Ethiopia in 2020 that found a higher proportion of maternal deaths (4.5%) were observed among women with preeclampsia compared to normotensive women (1%)^6^. Another study conducted in Ethiopia in 2019 revealed that the top three adverse maternal outcomes were reported as maternal death (2.8%), eclampsia (6.6%), and renal failure (1.1%)^7^. Also in 2019, HDPs were the third leading causes of maternal deaths in southern Ethiopia (16%), followed by obstetric hemorrhage (39%), and anemia (28%)^8^. The majority of maternal deaths occurred after the pregnant women developed convulsions and delays in care seeking behavior^9^.

The overall pooled prevalence of HELLP (Haemolysis, elevated liver enzymes, low platelet count) syndrome was 13% in 2019^3^. In southern Ethiopia, the main complications for admission were severe preeclampsia (51.8%), followed by postpartum hemorrhage (24.9%) in 2019. Eclampsia accounted for (70%) of pregnancy complications that occurred after 12 h of admission^8^. Women with severe preeclampsia have higher rates of organ dysfunctions, including kidneys, liver, brain, and the vascular system^7^.

A number of factors account for high rates of adverse maternal outcomes, including poor infrastructure, poor health-seeking behaviors and low socioeconomic conditions, shortage of supply and skilled manpower, weak referral systems, poor quality of care and access to timely obstetric care have all contributed to a higher proportion of maternal mortality in Ethiopia^10,11^.

The third Sustainable Development Goal plans to reduce the global maternal mortality rate to less than 70 per 100, 000 live births by 2030^12^. In line with this, the government of Ethiopia has a plan to reduce maternal mortality from 401 to 140 per 100,000 live births in 2030^13^. It has taken steps to strengthen engagement with key local and international sectors and stakeholders to address determinants of health^14^. Ethiopia recently replaced the previous four-visit focused antenatal care (ANC) model with the new ANC eight-contact model^14^. Furthermore, health facilities in Ethiopia have adopted a number of quality improvement measures such as providing 24-h services, including access to ambulances, and integrating the maternal deaths and near misses into regular practice to provide accurate information on causes of maternal deaths^11^.

Studies conducted in Ethiopia have not generated evidence that could be used by policymakers or practitioners because they did not include control groups or measure the risk of outcomes of interest and did not include socio-demographic variables such as maternal education status^8,15,16^. Another study done in southern Ethiopia was limited in estimating the risk of preeclampsia on adverse maternal outcomes because of poor ascertainment of exposures and outcomes using purposive sampling techniques^17^.

Moreover, this study’s findings will provide statistically valid epidemiological evidence for policy makers and implementers to reduce adverse maternal outcomes among women with preeclampsia and normotensive women. We aimed to measure the effect of preeclampsia on adverse maternal outcomes and identify risk factors among women with preeclampsia in Sidama region of southern Ethiopia.

## Results

### Socio-demographic and economic characteristics of study participants

**Figure 1:**
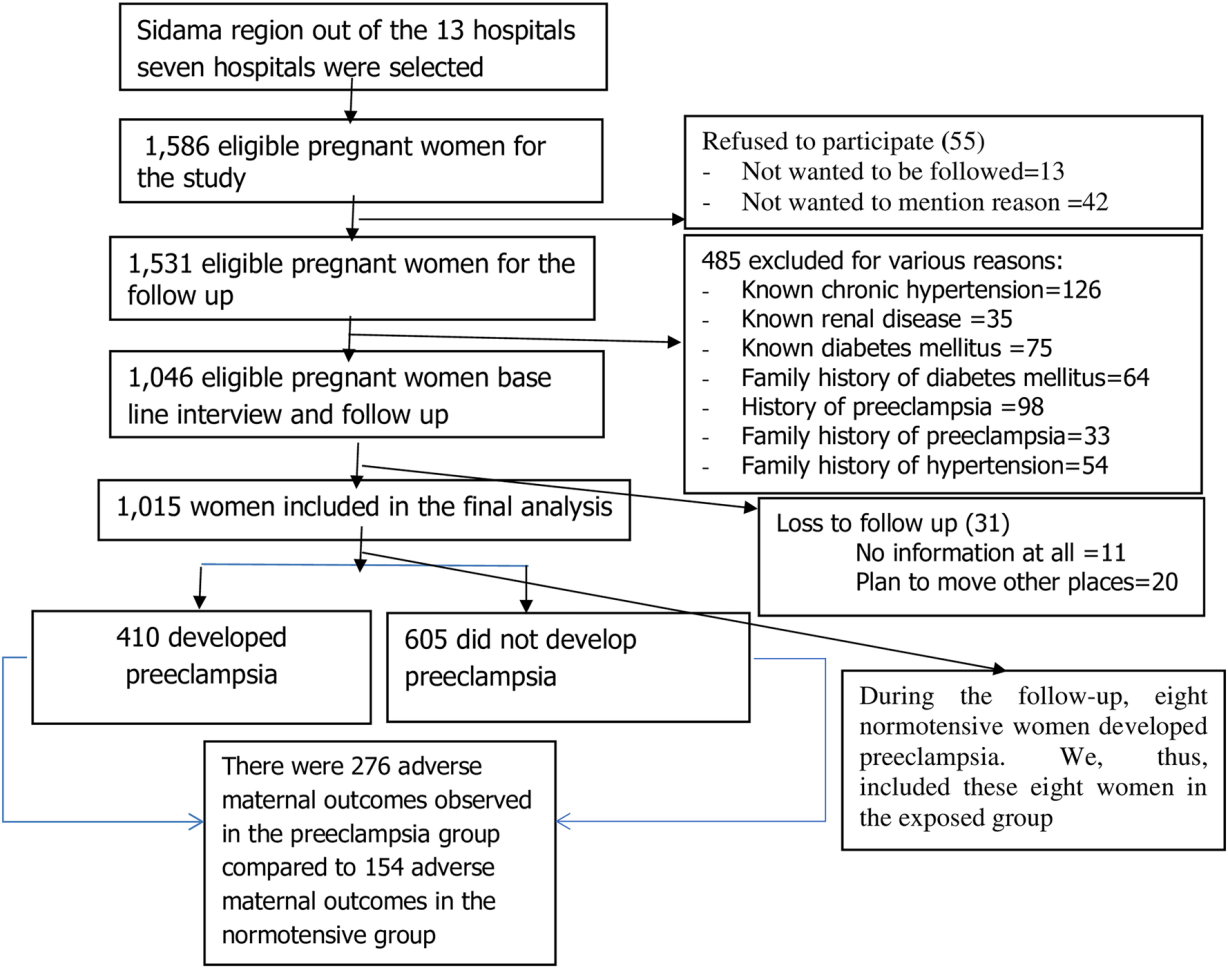
Flow-diagram of the overall study process in Sidama region, southern Ethiopia, August 8, 2019, to October 1, 2020.

We enrolled 1,015 pregnant women out of 1,586 pregnant women who were approached to participate in the study. Thirty-one (1.6%) of the participants were lost-to follow up. Of these, 10 were from the preeclamptic group and 21 from the normotensive group. Fifty-five women refused to participate in the study. During the follow-up, eight normotensive women developed preeclampsia. We, thus, included these eight women in the exposed group (Fig. 1).

The mean age of women with preeclampsia was 25.35 ± 4.76 years old, and 24.62 ± 4.55) for normotensive women. More than half (55.9%, 229/410) of women with preeclampsia were 16–24 years old compared to normotensive women (36.7%, 222/605), *P* < 0.001). Compared to normotensive women (4.5%, 27/605), a higher proportion of women with preeclampsia (10%, 41/410), *P* < 0.001) did not attend school. Compared to employed women (17.8%, 73/410) house wives had a higher proportion of preeclampsia (51.4% 211/410), *P* < 0.05) (Table 1).

**Table 1:** Socio-demographic and economic characteristics of women with preeclampsia and normotensive women in Sidama region, southern Ethiopia from August 8, 2019, to October 1, 2020.

Variables	Preeclampsia (n = 410)	Normotensive (n = 605)	Total (n = 1015)	*P* value(*)
**Age group (year)**	25.3[± 4.76]	24.62[± 4.55]		*P* < 0.001
16–24	229[55.9]	222[36.7]	451[44.4]	
25–34	167[40.7]	345[57]	512[50.4]	
≥ 35	14[3.4]	38[6.3]	52[5.1]	
**Maternal education**				
No formal education	41[10]	27[4.5]	68[6.7]	*P* < 0.001
Primary education	180[43.9]	225[37.2]	405[39.9]	
Secondary education	110[26.8]	201[33.2]	311[30.6]	
College/University	79[19.3]	152[25.1]	231[22.8]	
**Husband education**				
No formal education	22[5.4]	12[2]	34[3.3]	*P* > 0.05
Primary education	133[32.4]	145[24]	278[27.4]	
Secondary education	114[27.8]	186[30.7]	300(29.6)	
College/University	141[34.4]	262[43.3]	403[39.7]	
**Maternal occupation**				
House wife	211[51.4]	245[40.5]	456[44.9]	*P* < 0.05
Merchant	74[18]	139[23]	213[21]	
Employed	73[17.8]	149[24.6]	222[21.9]	
Farmer	12[2.9]	18[3]	30[3]	
Daily laborer	10[2.4]	16[2.6]	26[2.6]	
Student	30[7.3]	38[6.3]	68[6.7]	
**Husband occupation**				
Employed	129[31.5]	244[40.3]	373[36.7]	*P* > 0.05
Merchant	145[35.4]	225[37.2]	370[36.5]	
Farmer	83[20.2]	80[13.2]	163[16.1]	
Daily laborer	36[8.8]	30[5]	66[6.5]	
Unemployed	17[4.1]	26[4.3]	43[4.2]	
**Wealth index**				
Low	171[41.7]	163[26.9]	334[32.9]	*P* < 0.001
Middle	121[29.5]	216[35.7]	337[33.2]	
High	118[28.8]	226[37.4]	344[33.9]	
**Place of residence**				
Urban	107[26.1]	86[14.2]	193[19]	*P* < 0.001
Rural	303[73.9]	519[85.8]	822[81]	

*P value < 0.05 was considered statistically significant.

### Obstetric characteristics of women with preeclampsia and normotensive

A higher proportion of women with preeclampsia (45.6%, 187/410) was observed among women who were admitted at < 34 weeks compared to the normotensive group (10.9%, 66/605), *P* < 0.001). Compared to the normotensive group (36.5%, 221/605), a higher proportion of women with preeclampsia (41%, 168/410), *P* < 0.001) was observed in Yirgalem hospital. A higher proportion of cesarean delivery was observed among women with preeclampsia (45.1%, 185/410) compared to normotensive group (29.9%, 181/605), *P* < 0.001) (Table 2).

**Table 2:** Obstetrics characteristics of women with preeclampsia and normotensive women in Sidama region, southern Ethiopia from August 8, 2019, to October 1, 2020.

Variables	Preeclampsia (n = 410)	Normotensive (n = 605)	Total (n = 1015)	*P* value (*)
**Gravida**				
1	76[18.5]	114[18.8]	190[18.7]	*P* < 0.05
2–3	194[47.3]	363[60]	557[54.9]	
≥ 4	140[34.1]	128[21.2]	268[26.4]	
**Parity**				
Nulliparous	26[6.3]	12[2]	38[3.7]	*P* > 0.05
1	72[17.6]	111[18.3]	183[18]	
2–3	173[42.2]	406[67.1]	579[57]	
≥ 4	139[33.9]	76[12.6]	215[21.2]	
**Interpregnancy interval (IPI)**				
< 24 months (short (IPI)	22[5.4]	7[1.2]	29[2.9]	*P* > 0.05
24–59 months (optimal IPI)	182[44.4]	355[58.7]	537[52.9]	
60 + months (long IPI)	134[32.7]	132[21.8]	266[26.2]	
Not applicable (prim)	72[17.6]	111[18.3]	183[18]	
**Gestational age at admission (week)**				
< 34	187[45.6]	66[10.9]	253[24.9]	*P* < 0.001
34–37	223[54.4]	539[89.1]	762[75.1]	
**Antenatal care follow up in the past pregnancy**				
Yes	221[53.9]	412[68.1]	633[62.4]	*P* < 0.001
No	116[28.3]	80[13.2]	196[19.3]	
Not applicable (Prim pregnancy)	73[17.8]	113[18.7]	186[18.3]	
**Mode of delivery**				
Spontaneous vaginal delivery	193[47.1]	415[68.6]	608[59.9]	*P* < 0.001
Cesarean delivery	185[45.1]	181[29.9]	366[36.1]	
Vacuum assisted delivery	32[7.8]	9[1.5]	41[4]	
**Gestational age at delivery (week)**				
Extremely preterm (< 28)	11[2.7]	9[1.5]	20[2]	*P* < 0.001
Very preterm (28–32)	25[6.09]	2[0.63]	27[2.7]	
Moderate to late preterm (32–37)	112[27.3]	74[12.2]	186[18.3]	
Term + (≥ 37)	262[63.9]	520[86]	782[77]	
**Right upper quadrant pain/hepatomegaly**				
Yes	106[25.9]	3[0.49]	109[10.7]	*P* < 0.05
No	304[74.1]	602[99.5]	906[89.3]	
**Severe persistent vomiting and nausea**				
Yes	88[21.5]	3[0.5]	91[9]	*P* > 0.001
No	322[78.5]	602[99.5]	924[91]	
**Severe headache**				
Yes	221[53.9]	10[1.7]	231[22.8]	*P* < 0.05
No	189[46.1]	595[98.3]	784[77.2]	
**Hospitals**				
Adare general hospital	65[15.9]	190[41.4]	255[25.1]	*P* < 0.001
Hawassa referral hospital	57[13.9]	66[10.9]	123[12.1]	
Yirgalem general hospital	168[40]	221[36.5]	389[38.3]	
Hula primary hospital	11[2.7]	12[2]	23[2.3]	
Bona general hospital	55[13.4]	40[6.6]	95[9.4]	
Chuko primary hospital	16[3.9]	16[2.6]	32[3.2]	
Daye primary hospital	38[9.3]	60[9.9]	98[9.7]	

*P value < 0.05 was considered statistically significant.

### Incidence of adverse maternal outcomes among women with preeclampsia and normotensive women

There were 276 adverse maternal outcomes observed in the 410 preeclampsia group compared to 154 adverse maternal outcomes in the 605 normotensive group (*P* < 0.001). There were 4 maternal deaths were reported in the preeclampsia group compared to 0 deaths in the normotensive group (*P* < 0.05). Also, there were 53 antepartum hemorrhage was observed in the preeclampsia group compared to 24 antepartum hemorrhages in the normotensive group (*P* < 0.001). There were 90 acute kidney injury reported in the preeclampsia group compared to 12 acute kidney injury in the normotensive group (*P* < 0.001) (Table 3).

**Table 3:** Maternal outcomes among women with preeclampsia and normotensive women in Sidama region, southern Ethiopia from August 8, 2019, to October 1, 2020.

Variables	Preeclampsia (n = 410)	Normotensive (n = 605)	Total (n = 1015)	*P* value (*)
**Maternal death**				
Yes	4[1]	0	4[0.4]	*P* < 0.001
No	406[99]	605[100]	1,011[99.6]	
**Maternal intensive care unit admission**				
Yes	8[2]	1[0.2]	9[0.9]	*P* < 0.05
No	402[98]	604[99.8]	1006[99.1]	
**Antepartum hemorrhage**				
Yes	53[12.9]	24[4.0]	77[7.6]	*P* < 0.001
No	357[87.1]	581[96]	938[92.4]	
**Acute kidney injury**				
Yes	90[22]	12[2]	102[10]	*P* < 0.001
No	320[78]	593[98]	913[90]	
**Blood transfusion**				
Yes	29[7.1]	18[3]	47[4.6]	*P* < 0.05
No	381[92.9]	587[97]	968[95.4]	
**Postpartum hemorrhage**				
Yes	27[6.6]	17[2.8]	44[4.3]	*P* < 0.05
No	383[93.4]	588[97.2]	971[95.7]	
**Placenta abruption**				
Yes	9[2.2]	2[0.3]	11[1.1]	*P* < 0.05
No	401[97.8]	603[99.7]	1004[98.9]	
**Adverse maternal outcomes**				
Yes	276[67.3]	154[25.5]	430[42.4]	*P* < 0.001
No	134[32.7]	451[74.5]	585[57.6]	

*P value < 0.05 was considered statistically significant.

### Women with or without severity features of preeclampsia on adverse maternal outcomes and other risk factors

In the bivariable log-binomial logistic regression model, the following variables were identified as candidate variables for multivariable log-binomial logistic regression analysis: age of women, women without severe features of preeclampsia, women who were admitted to the hospital at < 34 weeks, women with severe features of preeclampsia, maternal education and occupation, husband’s education, place of residence, wealth index, mode of delivery, gravidity, parity, eclampsia, magnesium sulphate treatment, and antihypertensive treatment.

After controlling for confounders, we identified significant risk factors for adverse maternal outcomes as women with severe features of preeclampsia, women without severe features of preeclampsia, age, wealth status, and place of residence.

Women with severe features of preeclampsia had a 43% (aRR = 1.43, 95% CI 1.3–1.58) higher risk for adverse maternal outcomes compared to women without severe features of preeclampsia. Women without severe features of preeclampsia had a 39% (aRR = 1.39, 95% CI 1.2–1.76) higher risk for adverse maternal outcomes compared to women in the normotensive group. Women who were admitted to the hospital at < 34 weeks had a 25% (aRR = 1.25, 95% CI 1.1–1.46) higher risk for adverse maternal outcomes compared to women who were admitted between 34 and 37 weeks.

Women whose age group was 16–24 years old were 76% (aRR = 1.76, 95% CI 1.3–2.4) at a higher risk for adverse maternal outcomes compared to women whose age group was ≥ 35 years old. Women with preeclampsia whose age group was 25–34 years old were 73% [aRR = 1.73, 95% CI 1.2–2.3] at a higher risk for adverse maternal outcomes compared to women whose age group was ≥ 35 years old. Women from lower wealth quintiles were 40% [aRR = 1.40, 95% CI 1.3–1.70] more at risk for adverse maternal outcomes compared to women from higher wealth quintiles. Women who resided in rural areas were 24% [aRR = 1.24, 95% CI 1.2–1.5] at risk for adverse maternal outcomes compared to women who resided in urban areas (Table 4).

**Table 4:** A multivariable log-binomial logistic regression model for risk factors for adverse maternal outcomes among women with preeclampsia in Sidama region, southern Ethiopia from August 8, 2019, to October 1, 2020.

Variables	Adverse maternal outcomes		cRR^‡^ (95%Cl^†^)	aRR^||^ (95%Cl^†^)
	Yes (%) (n = 430)	No (%) (n = 585)		
**Gravidity**				
1	83[43.7]	107[56.3]	0.7**[0.6–0.8]	0.5[0.4–1.2]
2–3	218[39.1]	339[60.9]	1.0[0.8–1.3]	1.12[0.5–1.8]
≥ 4	129[48.1]	139[51.9]	1	1
**Parity**				
Nulliparous	23[60.5]	15[39.5]	0.8[0.4–1.3]	0.6[0.5–1.4]
1	73[39.9]	110[60.1]	0.6*[0.6–0.9]	0.8[0.4–1.4]
2–3	223[38.5]	356[61.5]	1.1[0.8–1.3]	1.06[0.76–1.55]
≥ 4	111[51.6]	104[48.4]	1	1
**Age of women (year)**				
16–24	229[50.8]	222[49.2]	1.3[0.7–1.5]	1.76**[1.3–2.4]
25–34	180[35.2]	332[64.8]	1.6*[1.4–1.9]	1.73**[1.2–2.3]
≥ 35	21[40.4]	31[59.6]	1	1
**Maternal education**				
No formal education	39[57.4]	29[42.6]	1.02[0.8–1.4]	1.2[0.6–1.3]
Primary education	203[50.1]	202[49.9]	0.8*[0.7–0.9]	0.6[0.5–1.3]
Secondary education	103[33.1]	208[66.9]	0.8[0.7–1.1]	0.7[0.5–1.2]
College/University	85[36.8]	146[63.2]	1	1
**Mode of delivery**				
SVD^††^	243[39]	365[60]	1	1
Cesarean delivery	162[44.2]	204[55.7]	3.8***[3.2–4.4]	3.96[0.65–4.7]
Vacuum assisted	25[61]	16[29]	1.3[0.5–3.4]	1.3[0.6–3.9]
**Husband education**				
No formal education	20[58.8]	14[41.2]	1.02[0.7–1.5]	1.1[0.6–1.9]
Primary education	134[48.2]	144[51.8]	0.9[0.8–1.1]	0.91[0.8–1.4]
Secondary education	131[43.7]	169[56.3]	0.8[0.3–1.3]	0.9[0.6–1.6]
College/University	145[36]	258[64]	1	1
**Maternal occupation**				
House wife	208[45.6]	248[54.4]	1.9[0.7–2.1]	1.6[0.6–2.3]
Merchant	83[29]	130[61]	2[0.80–3.2]	1.2[0.8–1.6]
Employed	80[36]	142[64]	1	1
Farmer	20[66.7]	10[33.3]	1.1[0.6–1.4]	1.0[0.5–1.8]
Daily laborer	11[42.3]	15[57.7]	1.2[0.8–1.8]	1.4[0.6–2.4]
Student	28[41.2]	40[58.8]	0.9[0.7–1.1]	0.8[0.6–1.5]
**Wealth index**				
Low	176[52.7]	158[47.3]	1.4*[1.2–1.6]	1.40**[1.3–1.7]
Middle	143[42.4]	194[57.6]	1.2[0.9–1.4]	1.3*[1.2–1.6]
Rich	111[32.3]	233[67.7]	1	1
**Women with severe features of preeclampsia**				
Yes	112[88.9]	14[11.1]	1.44***[1,2–1.70]	1.43**[1.3–1.58]
No	318[35.8]	571[64.2]	1	1
**Place of residence**				
Urban	107[55.4]	86[44.6]	1	1
Rural	323[39.3]	499[60.7]	1.16*[1.1–1.4]	1.24**[1.2–1.5]
**Gestational age at admission (week)**				
< 34	154[60.9]	99[39.1]	1.39***[1.2–1.61]	1.25**[1.1–1.46]
34–37	276[36.2]	486[63.8]	1	1
**Women without severe features of preeclampsia**				
Yes	276[67.3]	134[32.7]	1.42***[1.13–1.8]	1.39**[1.2–1.76]
No	154[25.5]	451[74.5]	1	1
**Eclampsia**				
Yes	29[90.6]	3[9.4]	1.45*[1.3–1.85]	1.34[0.56–1.63]
No	401[40.8]	582[59.2]	1	1
**Magnesium sulfate treatment**				
Yes	175[62.7]	104[37.3]	0.80**[0.70–0.92]	0.90[0.69–1.18]
No	255[34.6]	481[65.4]	1	1
**Antihypertensive treatment**				
Yes	196[63]	115[37]	0.75*[0.64–0.87]	0.84[0.65–1.10]
No	234[33.2]	470[66.8]	1	1

^†^CI = Confidence interval.

^‡^cRR = Crude relative risk.

^††^SVD = Spontaneous vaginal delivery.

^||^aRR = ADJUSTED relative risk.

^¶^1-Reference group.

*P < 0.05; **P < 0.001; ***P < 0.0001.

## Discussion

In this study, higher level of adverse maternal outcomes was observed in the preeclampsia group compared to normotensive group in the Sidama region after controlling for confounders. Maternal death, maternal ICU admission, postpartum hemorrhage, antepartum hemorrhage, and blood transfusion were higher in the preeclampsia group compared to the normotensive group. We identified significant risk factors for adverse maternal outcomes such as women who had severe features of preeclampsia, who were admitted to hospital at < 34 weeks of gestations, with low wealth status, younger women and who lived in rural areas.

More women with preeclampsia had adverse maternal outcomes compared to normotensive women. Our finding was higher than another prospective study in Uganda in 2020 that found 19.4% of adverse maternal outcomes^18^ and higher than a 2018 South African study that found 17.6% had kidney injury^19^. The variation among studies regarding incidence proportion of adverse maternal outcomes has been linked to the season the study was conducted in the severity of preeclampsia, and gestational age at diagnosis.

More maternal deaths were observed among women with preeclampsia compared to normotensive women in our study. This finding was lower than another study in Ethiopia in 2015 that found that (5%) of mothers died^9^, but lower than another study in Uganda in which 20 mothers died^20^. Raising awareness at community level among mothers during ANC visits regarding early signs of adverse outcomes and informing them of the referral system could help reduce delays in accessing treatment.

Maternal ICU admission was higher in the preeclampsia group compared to normotensive group. This finding was lower than the finding of another study conducted in the Brazil in 2014 (74.2% vs 21.9%)^21^, in Ghana (10.7%)^22^, and in Uganda (3%)^23^. This might due to poor knowledge of the communities on emergency obstetric conditions and their management and readiness among health facilities and preference for cultural remedies. Educating women on the importance of attending ANC services and the advantages of using such services can reduce maternal mortality. Understanding the physical and psychological stress that might make a woman at risk of the occurrence of preeclampsia could improve attendance.

Higher level of postpartum hemorrhage was reported in the preeclampsia group compared to normotensive group. This finding was supported by other studies^21,24^. Postpartum bleeding was more frequent in women with preeclampsia (22.9%) compared to normotensive women (13.9%)^24^. This finding was higher than another study conducted in Kenya in 2020 (4.4 versus 3.7%)^25^. This variation might be due to the introduction of magnesium-sulphate to treat women who have severe preeclampsia/eclampsia, thus leading to a reduction of incidence proportion of postpartum hemorrhage.

Higher level of antepartum hemorrhage was observed in the preeclampsia group compared to normotensive group. This finding was higher than another study done in Ethiopia in 2011 (5.1%) of APH^26^, and in Nigeria in 2020 (3.5%) of APH^27^. This could be because of women with severe preeclampsia associated with uterine blood vessel problems and higher risk for arterial dysfunction^28^. It is important to re-design and re-package existing interventions in the region that focus on improving ANC services, screening of elevated blood pressure and proteinuria in early pregnancy.

More women with preeclampsia had blood transfusions compared to normotensive women. This finding was similar with other studies conducted in Brazil in 2014, which showed that a higher proportion of blood transfusion was reported among women with preeclampsia compared to women in the normotensive group (52.9% vs 7.9%)^21^ and in southern Ethiopia in 2019 (57.8%)^8^. This could be because severe preeclampsia is related to a significant cause of cesarean delivery and cesarean delivery is also related to excessive blood loss which can be due to underlying preeclampsia-induced coagulopathy as well^24^. One of the important contributing factors to high maternal mortality in low-income countries is the late arrival to hospital at an irreversible stage of the disease^29^. It is essential at the health facility level to design a better tracking system for pregnant women for ANC visits and provide health facilities with MgSO4, which could prevent convulsions and severe outcomes in mothers.

Women with severe features of preeclampsia had a higher risk for adverse maternal outcomes compared to women without severe feature of preeclampsia. This finding was similar to a several other studies^30–32^. However, this finding was also lower than a study in Uganda in 2020 that found women with severe preeclampsia had higher rates of (93.2%) of adverse maternal outcomes compared to those without severe preeclampsia^18^. This could be because the women accessed ANC visits too late or too near their delivery date. The increased risk of maternal complications that was observed might be explained by the progression of preeclampsia to severe features and women who developed preeclampsia before 34 weeks^30^. Thus, the maternity care providers should ensure that the pregnant woman and her family are aware of the common dangerous symptoms and signs related to preeclampsia and are ready to act without delay to seek care in a health care facility.

Women who were admitted to the hospital at < 34 weeks had a higher risk for adverse maternal outcomes compared to women who were admitted between 34 and 37 weeks. This finding was supported by another study^33^ showed the early onset of preeclampsia was associated with an increased risk for adverse maternal outcomes compared to late-onset of preeclampsia^33^. Early-onset of preeclampsia is associated with severe placental dysfunction and fetal growth restriction compared to late-onset of preeclampsia^33^. Thus, maternal health care providers should be aware of screening and identifying high-risk pregnant women with respect to preeclampsia, which is used for timely initiation of appropriate management to reduce further adverse maternal complications.

Women with low wealth status were at higher risk for adverse maternal outcomes compared to women with a high wealth status. This finding was supported by another study in Zimbabwe in 2019 found that women having a low wealth status were a higher risk for adverse maternal outcomes^37^. This finding was supported by another study in Ethiopia the wealthiest women were 2 times more likely to have ANC visits than women in the poorest quintile^38^. This could be due to adolescent age group where the endocrinological and the immunological systems are yet immature and also due to poor nutrition.

Younger women were at higher risk for adverse maternal outcomes compared to older women. This finding was agreed with another study^34^. Younger, as evidence suggests that teenaged women are more likely to develop preeclampsia. Also, the ones who conceive immediately after coitarche too are at risk^34^. Women, who are younger or lower socioeconomic status, may be vulnerable to poorer quality of care and mistreatment during childbirth. This finding is contradicted with other studies study conducted in Tanzania and Ethiopia found that mothers aged > 35 years old had 2.6 and 2 times more likely to develop preeclampsia compared to younger mothers^35,36^. The variation among studies might be due to differences in marriage culture, believe and religious teachings on marriage which varies from one country to another and age categorization among studies also varied.

Women who resided in rural areas were higher risk for adverse maternal outcomes compared to women who reside in urban areas. This finding was similar with another study in Ethiopia^29^ found that women who lived in rural areas were might be a lower socioeconomic status than urban residents, which could lead to less health-seeking behavior. Low health-seeking behavior among pregnant women makes them less likely to attend antenatal care clinics, resulting in a delay in diagnosis and treatment associated with preeclampsia^29^. Other study in Ethiopia in 2022 showed that the odds of optimal ANC visit is 42% lower in rural women compared to women living in urban areas^38^. There is a need to focus on awareness creation on antepartum and intrapartum care and risk preparedness so rural women can seek care when they experience preeclampsia symptoms.

One limitation could be recall bias linked to gestational age, which was calculated based on the women’s recall of their last menstrual period. However, women who could not remember the approximate gestational age were given an ultrasound scan. Social desirability could have been present because data were collected in face-to-face interviews, which could have led to socially acceptable answers. This study is not generalizable as it was limited to one region of the country, and it was limited to women who received hospital care. It also measured short-term adverse maternal outcomes and the development of preeclampsia in among pregnant women, but it did not assess the risk of preeclampsia in later life, the risk of chronic hypertension, chronic renal failure, venous thromboembolism, ischemic heart disease, and cardiovascular events in later life, or other important health outcomes. However, it is unlikely that this limitation substantially affected our results. Because our study focused on one region and so is not representative of the whole country, but we carefully identified the potential sources of bias early in the design, validated the tool before data collection, and the percentage of pregnant women engaged in the study. Furthermore, this study has enabled us to identify the effect of preeclampsia on adverse maternal outcomes and risk factors in our setting, which can be used to predict and be used as a source of information in the whole country of Ethiopia in a similar socioeconomic setting.

## Conclusion

In this study, more adverse maternal outcomes occurred among women with preeclampsia after controlling for confounders. A higher risk of maternal outcomes was observed among women with preeclampsia, especially among women with severe features of preeclampsia, and those admitted to hospital at < 34 weeks. This paper highlights the significantly elevated maternal risks associated with pre-eclampsia, especially when it has severe features. These effects could be detected and controlled early in pregnancy. Other risk factors for adverse maternal outcomes were identified as younger women, with low wealth status, and who lived in rural areas. Healthcare providers should raise awareness of preeclampsia in antenatal care clinics, focusing on younger women, low wealth status, and those living in rural areas.

### Implications for future research

This study provides epidemiological evidence for the effect of preeclampsia on adverse maternal outcomes in clinical and public health practices. We recommend early screening of preeclampsia during pregnancy to facilitate more timely referral and initiation of early treatment of severe features of preeclampsia. Furthermore, a large cohort study should be conducted to evaluate other types of hypertensive disorders of pregnancy on the incidence of adverse maternal outcomes.

## Methods

### Study design and setting

A prospective open cohort study was conducted from August 8, 2019, to October 1, 2020 in the Sidama region of Ethiopia. In 2020, the population of the region was approximately 4 million. At that time, there were thirteen public hospitals, 138 health centers, and 540 health posts providing maternal, newborn, and child health services. In 2020, approximately 132,031 pregnant women attended ≥ 4 antenatal care visits (ANC), and 127,585 births were assisted by skilled birth attendants. Out of the 13 hospitals that are found in the region, we enrolled participants from seven of the hospitals, including Adare, Hawassa, Yirgalem, Hula, Bona, Chuko, and Daye hospitals.

### Study population

The participants of this study were women with preeclampsia and normotensive women who were enrolled at ≥ 20 weeks of gestation until the 37th week and they were followed until 42 days after delivery the enrolled woman’s outcome status was ascertained. During the follow-up, 253 women with preeclampsia and normotensive women were admitted to the hospitals at < 34 weeks of gestation, and 762 women with preeclampsia and normotensive women were admitted to the hospitals at 34–37 weeks of gestation.

### Eligibility criterion

#### Inclusion criteria

Pregnant women with hypertension plus proteinuria, mild hypertension and evidence of organ dysfunction, severe hypertensive without proteinuria, and evidence of organ dysfunction were included in the study^39,40^. Pregnant women with preeclampsia and normotensive women were selected by health care providers: general medical practitioners, emergency surgical officers, or obstetricians/gynecologists during the follow-up.

#### Exclusion criteria

Pregnant women who had known chronic hypertension before 20 weeks of gestation or women who were already on antihypertensive medication, women who had known chronic renal disease, known history of diabetes mellitus, known history of preeclampsia, family history of diabetes mellitus, family history of preeclampsia, and family history of hypertension were excluded from the study^41,42^.

#### Operational definitions

The diagnosis of adverse maternal outcomes was supported by guidelines of the Obstetrics Management Protocol for Hospitals in Ethiopia and was based on the recent International Society for the Study of Hypertension in Pregnancy in 2021^39,40^. Antepartum hemorrhage was defined as vaginal bleeding during labor beginning in the 28th week of pregnancy^39^. Postpartum hemorrhage was defined as vaginal bleeding lasting more than 500 ml after childbirth and was diagnosed by any of the following: visual estimation, 10% reduction of hemoglobin from antepartum, a blood loss that needs transfusion^39^.

Acute kidney injury (AKI) was defined as a syndrome characterized by a rapid (hours to days) deterioration of kidney function or was a clinical syndrome characterized by an abrupt decline in glomerular filtration rate and the accumulation of nitrogenous waste products. AKI was defined as an increase in serum creatinine of 0.3 mg/dl within 48 h or in urine volume of 0.5 ml/kg/hr for 6 h. Although AKI diagnosis also depends on the decision-making process of the clinician rather than underlying renal function^43^. Maternal ICU admission was defined as admission to a critical care area providing at least additional monitoring and interventions for women who had severe complications^44^. Maternal death was defined as the death of a woman while pregnant or within forty-two completed days of termination of pregnancy, irrespective of duration and site of pregnancy, from any cause related to or aggravated by the pregnancy or by its management but not due to accidental or incidental causes^40^.

#### Sample size and sampling

The sample size was calculated using EPINFO version 7. We considered the following assumptions for sample size calculations: women who had anemia^45^, the ratio of exposed to unexposed group (1 to 1), the proportion of anemia among women with preeclampsia was 10.835%, the proportion of anemia among normotensive women was 16%. The sample size was estimated to be 1,586 cohort of pregnant women were enrolled, accounting for a design effect of two and 10% loss to follow-up. We also assumed a two-sided confidence level of 95% with a power of 80%.

A two-stage cluster sampling techniques were used to recruit study participants. In the first stage, seven of the thirteen hospitals were selected using the simple random sampling technique. In the second stage, women with preeclampsia and normotensive women and maternal conditions were selected from the cohort of pregnant women using a cluster sampling technique.

#### Exposure ascertainments

We ascertained exposure of interest supported by guidelines of the Obstetrics Management Protocol for Hospitals in Ethiopia in 2021 and on the recent International Society for the Study of Hypertension in Pregnancy^39,40^. The main exposure variable for this study was preeclampsia (preeclampsia with or without severity features). Preeclampsia was defined as the presence of proteinuria (≥ 1 + or 0.3 g/L) and hypertension (≥ 140/90 mmHg) on two occasions at least 4 h apart detected after the 20^th^ week of gestation in a previously normotensive woman. Preeclampsia without severe features was defined as one or more of the following conditions: absence of systemic involvement and raised BP ≥ 140/90 mmHg plus 24-h urine protein ≥ 300 mg/24 h or urine dipstick >  + 1after 20 weeks of gestation in previously normotensive women. Preeclampsia with severe features was defined as one or more of the following conditions: BP ≥ 160/110 mmHg, hepatic dysfunction, pulmonary edema and/altered mental status, headache, blurred vision, right upper quadrant pain, blindness, seizures and disseminated intravascular coagulation, and elevated liver enzymes^39^. Eclampsia was diagnosed as the presence of convulsions that could not be attributed to other causes in a woman with preeclampsia. Normotensive women were pregnant women having a (BP) < 140/90 mmHg with ≥ 20 weeks of gestation or who did not develop preeclampsia and proteinuria. Gestational age was calculated based on a woman’s recall of her last menstrual period. However, an ultrasound scan was used for those women who could not remember their last menstrual period^39^.

#### Outcome variable

Adverse maternal outcomes were defined as the occurrence of at least one of the following conditions: maternal death, maternal ICU admission, blood transfusion, postpartum hemorrhage, acute kidney injury and antepartum hemorrhage.

#### Data collection

Before data collection, we validated the data collection tool^46^. Two bilingual translators (speakers of both Sidamic and English languages) were selected to translate the information into the Sidamic language in a way that more accurately reflected the tone of the language. The translations were compared and discrepancies were noted during the translation process. Poorer wording choices were identified and resolved in a discussion between the translators.

The back translations were done by two experts in the source language (English). Face and content validation of the tool was done by a panel of experts (midwife experts, epidemiologists, and gynecologists). The panel of experts independently assessed the tool for readability, intelligibility, clarity, and ease of use. The internal consistency for each dimension was checked using Cronbach’s alpha (Cronbach’s alpha = 0.98).

In the first pilot test, conducted in a non-study area, all participants responded to all items in the data collection tool and marked them correctly. No missing items were found. Data collectors also reported no difficulty in asking the questions, and no participant reported having any problem understanding the items. The tool was tested for the second time two weeks after the first measurement. The two-week test–retest reliability result was shown to have a good correlation with reliable strategies to assess these point scores (Intraclass Correlation Coefficients (ICC) for agreement of 0.78; *P* < 0.001) because the ICC value was found to be in the range of 0.75 to 0.9, indicating good reliability^47^.

Trained midwives conducted face-to-face interviews at antenatal care clinics using the pre-tested validated tool. Besides, a checklist was used to collect information from the maternal records of women with preeclampsia and normotensive women in each hospital. We collected socio-demographic information and clinical and laboratory variables linked to maternal and perinatal outcome status. The data collection procedures were supervised by three Maternal and Child Health maternity and reproductive health professionals.

#### Outcome ascertainments

Adverse maternal outcome was ascertained by an obstetrician and gynecologist and trained midwives. Client medical registration was also used to retrieve adverse maternal outcome status. For those discharged, maternal condition was ascertained on the postnatal care follow up appointment and those who did not show up for this follow up, phone call was used.

### Confounders ascertainment

#### Covariates

These confounders were identified by prior theoretical knowledge and literatures. Potential confounding variables are those variables that have an association with an exposure variable and the outcomes. The following covariates were considered as possible confounders: Women who developed preeclampsia differed in maternal age, gravidity, history of chronic hypertension (personal or family), personal history of preeclampsia, history of diabetes, and family history of preeclampsia from those who did not develop preeclampsia (*P* > 0.05)^48–51^.

#### Strategy to control confounders at the design stage

When pregnant women disappear from either the exposed or non-exposed group or both, might bias be introduced? In our study, pregnant women's loss means dropping out of the follow-up because of death or complications during ANC visits, which was considered as missing completely at random. We used the following solution to minimize it. Maternal condition was determined for those discharged to home at the postnatal care follow-up appointment, and those who did not show up for this follow-up were contacted by phone. To minimize misclassification bias, consideration was given at the design stage and by using different sources of information for exposure and outcome ascertainment. In those who couldn’t recall their last normal menstrual period, gestation age was estimated by using ultrasound. To minimize confounds, study restrictions were implemented during the design stage.

### Statistical analysis

Data were cleaned, coded and analyzed using Stata 14. We identified outliers and missing values and checked data consistency using the original questionnaire for the responses using participants’ code numbers. Mean and standard deviations were computed for continuous variables. Frequencies and percentages were computed for categorical variables. An incidence proportion of adverse maternal outcomes were conducted on women who had preeclampsia and normotensive women. Cross tabulation was also performed to test the relationship of exposure variables with the outcome variable. A chi-squared test was used to compare categorical variables between women with preeclampsia and normotensive women.

Principal component analysis was computed and used for wealth index computation and was ranked into three groups as low, middle, and high. A composite measure of the household's cumulative living standard was calculated by using data on household ownership of selected assets, like various household assets and means of transportation. Different items for urban and rural areas were computed separately. We included 21 items for rural residents and 16 items for urban residents. The suitability of data was computed by using Bartlett’s test of Sphericity and the Kaiser–Meyer–Olkin (KMO) measure of sample adequacy^52^. The KMO > 0.6 was used to confirm the sample adequacy for factor analysis^52^.

A multivariable log-binomial logistic regression model was performed to identify the risk factors for adverse maternal outcomes. According to Hosmer and Lemeshow, a variable with a *P* value < 0.25 was recommended as a screening criterion for the selection of candidate variables used in a multivariable log-binomial logistic regression model^53^. This confirmed that insignificant variables from the first step were reanalyzed in later steps. Moreover, the candidate variables were also considered based on the subject matter expertise of professionals such as gynecologists, obstetricians, epidemiologists, and statisticians who were working as supervisors and who provided more subject matter expertise to improve the modeling process substantially. Indeed, this insight from subject matter experts substantially improved the modeling process^53^. A variable with a *P* value of < 0.05 was used to identify statistically significant risk factors for adverse maternal outcomes. Relative risk with a 95% confidence interval was reported.

We checked for multicollinearity among contributing factors using a variance inflation factor at a cutoff point of ten^54^. We confirmed that there was no collinearity among predictors. The goodness of fit was tested using the Hosmer–Lemeshow test. The predictor that was greater than the significance level (*P* value > 0.05) was accepted^55^. This indicates that the observed model did not significantly differ from the expected model.

### Ethical considerations

This study was reviewed and ethical approval was issued by the Institutional Review Board of the University of Gondar with R.No.O/V/P/RCS/044/2019 in March 2019. All participants signed an informed consent document before study participation began. Pregnant women having abnormal clinical and laboratory results were referred for treatment. Women with severe hypertension were provided with antihypertensive treatment; those with convulsions were given appropriate treatment. We confirmed that all methods were carried out in accordance with relevant guidelines and regulations.

## Data Availability

The data that supports the findings of this study is available from the corresponding author upon reasonable request in the form of Stata Version 14.
